# A Case of Congenital Rubella Syndrome in the Netherlands: A Brief Report on Rubella Virus Surveillance

**DOI:** 10.1097/INF.0000000000004849

**Published:** 2025-05-21

**Authors:** Laurentia F. Loeve, Vasiliki-Lydia Sideridou, Elisabeth H. Schölvinck, Ruben B. Brandsema, Coretta C. van Leer-Buter, Xuewei Zhou

**Affiliations:** From the * Department of Neonatology, Beatrix Children’s Hospital; † Department of Medical Microbiology; ‡ Department of Pediatric Infectious Diseases/Immunology, Beatrix Children’s Hospital, University of Groningen, University Medical Center Groningen, Groningen, The Netherlands.

**Keywords:** rubella virus, congenital rubella syndrome, rubella vaccine, rubella surveillance, measles

## Abstract

A Somalian asylum seeker gave birth in the Netherlands to a full-term neonate with congenital rubella syndrome. The mother was probably in Somalia or en route to Europe during her infectious period. Given the worldwide ongoing humanitarian crises in regions of low vaccination coverage, awareness in countries receiving refugees is needed together with ongoing rubella vaccine campaigns in immigrant populations.

The World Health Organization (WHO) has accelerated rubella elimination since 2012 through universal introduction of a rubella vaccine in all countries.^1^ During pregnancy, primary rubella virus infection may lead to miscarriage or fetal death, or cause a variety of organ defects known as congenital rubella syndrome (CRS) in live births.^2^

By 2022, the WHO had introduced the rubella-containing vaccine (RCV) in 175 of 194 (90%) countries which has led to an increase of RCV first dose coverage in children. However, low-income countries still have the lowest coverage rates, and adults, more specifically women of childbearing age, are yet not well protected.^1^

## CASE REPORT

In May 2024, a female asylum seeker from Somalia who had registered a month earlier in a refugee center in the Netherlands gave birth to a full-term female neonate. She had not received medical care up to 33 weeks of gestation when she arrived in the Netherlands. The neonate was born with a remarkable diffuse purpura on her face and body, known as “blueberry muffin” syndrome. Laboratory results showed thrombopenia (16 × 10^9^/L) with a high number of immature platelets (28.6%) for which 2 thrombocyte transfusions were given. Physical examination revealed the presence of hepatosplenomegaly and respiratory distress for which continuous positive airway pressure was initiated. The differential diagnosis of blueberry muffin syndrome in babies includes TORCH infections (toxoplasmosis, others, rubella, cytomegalovirus and herpes simplex virus) and malignancies (leukemia and neuroblastoma). Appropriate investigations did not show signs of malignancy. Urine and serum samples were examined for an infectious cause. Urine tested positive by reverse transcription polymerase chain reaction for rubella virus and the child’s serology showed positive rubella IgM and IgG titers. The mother was also tested for rubella virus antibodies and showed a very high serum IgG but negative IgM; her urine tested reverse transcription polymerase chain reaction negative for rubella virus. The mother reported having a facial rash during the fifth month of pregnancy without any other symptoms. This may have marked the timing of the infection, at which point in time she was still in Somalia. The mother does not recall being vaccinated against rubella.

Given the diagnosis of CRS, the neonate was screened for other CRS abnormalities. Echocardiogram, cerebral ultrasound, brain magnetic resonance imaging, comprehensive hearing screening by the audiologist and ophthalmologic examination by the ophthalmologist revealed no abnormalities continuous positive airway pressure could be discontinued on day 3. Thrombocyte count was monitored daily. On day 4 and day 8, platelet levels again decreased, necessitating the administration of a new platelet transfusion.

Strict infection prevention measures (contact-droplet isolation) were applied in the neonatal ward. Children with CRS can be a major source of infection due to the prolonged shedding of the virus (months-years) in pharyngeal secretions and urine.^3^ Infection prevention precautions were also given to the refugee center. Other immigrants from countries are at risk for infection since they may not have been vaccinated and the risk for prenatal vertical transmission is high in nonimmune pregnant women.

### Current Rubella Virus Surveillance in the Netherlands, Europe and Worldwide

In the Netherlands, the last reported rubella case before our patient dates from 2015. RCV was introduced in the national Dutch vaccination schedules in 1987.^4^ Since 2000, the reported rubella outbreaks were in unvaccinated school children from communities that do not vaccinate their children for religious reasons.

In Europe, 29 countries routinely report rubella data to the European Centre for Disease Prevention and Control. From February 1, 2024 to January 31, 2025, these countries reported a total of 224 cases of rubella, of which only 28 (1.0%) were laboratory-confirmed.^5^ Confirmed infections were reported by Germany (n = 13), Poland (n = 7), Sweden (n = 3), Latvia (n = 2), France (n = 1), Lithuania (n = 1) and Italy (n = 1). The European Centre for Disease Prevention and Control has no details on CRS cases.^5^ According to the WHO, the estimated vaccination coverage in the European region is 95% in 2023.^6^

Worldwide the number of reported rubella decreased by 97% between 2000 and 2022. Nonetheless, the number of cases is still high. In 2022, a total of 17.407 rubella cases and 1.527 CRF cases were reported. The highest number of rubella cases were found in the African and South-East Asia Region. However, the African and Eastern Mediterranean regions reported the lowest rubella vaccination coverage (36% and 42%, respectively).^1^ Discrepancies between the number of rubella cases and the vaccination coverage in the African and Eastern Mediterranean Region may be due to inadequate reporting.

**FIGURE 1.**
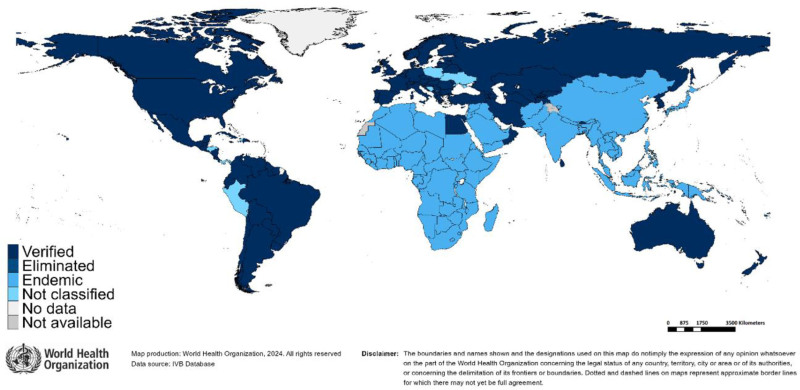
State of rubella eradication, rubella elimination data available at WHO HQ (Geneva) as of March 2025 (https://immunizationdata.who.int/).

Figure 1 illustrates the current endemic regions of rubella as well as those where it has been eliminated. The only WHO region where rubella elimination through vaccination has been officially achieved is the region of the Americas (AMR). AMR eliminated rubella in 2009.^7^ In the United States, rubella has been eliminated since 2004. Many rubella cases are observed in individuals who have been infected outside of the United States.^8^

## DISCUSSION

Since 2015, no rubella cases have been reported in the Netherlands. Likewise in Europe, the rubella notification rate is zero in most countries except for the cases. Vaccine-preventable diseases are increasing, however, as currently observed for measles. Between February 1, 2024 and January 31, 2025, 30 EU/EEA Member States reported a total of 32,265 measles cases of which 22,961 laboratory confirmed.^5^ Both rubella and measles, can be prevented by a single vaccine, which also prevents mumps. Although this combined vaccine measles, mumps, rubella (MMR) is recommended by the WHO, some countries still offer single vaccines for these diseases, which delays protection against all 3 viruses and leaves a risk that schedules are not completed. There are still 19 countries where the rubella vaccination has not been introduced. Four are in the Eastern Mediterranean Region and 15 are in the African Region (including Somalia).^9^

Unvaccinated populations at risk for measles may also be at risk for rubella. These 2 viruses have distinct clinical features, but initial symptoms may be very similar. Both diseases can resemble a cold with symptoms of rash and fever. As noted above, laboratory confirmation is not always performed before notification. In July 2023 for example, Somali Red Crescent Society volunteers reported rash and fever, in individuals testing negative for measles. Rubella virus was detected in some of their samples.^10^ The children from whom the samples were taken had only been vaccinated against measles because the combined MMR vaccine was not available at the time. Therefore, a vaccination campaign with the combined MMR vaccine is planned for 2024.

In our specific patient, the mother was probably still in Somalia during her infectious period. Because there is no certainty as to when the mother experienced the infection, it is conceivable that she infected other susceptible individuals, either in Somalia or in route to Europe. Given the worldwide ongoing humanitarian crises in regions of low or no vaccination coverage, the whole world needs to be aware of potential outbreaks, including those from the rubella virus. Vigilance is warranted, which means increasing awareness and preparedness, including ongoing vaccine campaigns in immigrant populations. Laboratory confirmation of measles and rubella remains important since these 2 diseases can present with similar symptoms. Rubella, however, can lead to CRS in newborns with a signature of possible birth defects.
